# Properties of Ramie (*Boehmeria nivea* (L.) Gaudich) Fibers Impregnated with Non-Isocyanate Polyurethane Resins Derived from Lignin

**DOI:** 10.3390/ma16165704

**Published:** 2023-08-20

**Authors:** Vincentius Yolanda Angger Raditya, Muhammad Adly Rahandi Lubis, Rita Kartika Sari, Petar Antov, Seng Hua Lee, Lubos Kristak, Efri Mardawati, Apri Heri Iswanto

**Affiliations:** 1 Department of Forest Products, Faculty of Forestry and Environment, IPB University, Bogor 16680, Indonesia; anggerraditya@gmail.com; 2 Research Center for Biomass and Bioproducts, National Research and Innovation Agency, Jakarta Pusat 16911, Indonesia; 3 Research Collaboration Center for Biomass and Biorefinery between BRIN and Universitas Padjadjaran, Bandung 45363, Indonesia; efri.mardawati@unpad.ac.id; 4 Faculty of Forest Industry, University of Forestry, 1797 Sofia, Bulgaria; p.antov@ltu.bg; 5 Department of Wood Industry, Faculty of Applied Sciences, The MARA Technological University, Shah Alam 40450, Malaysia; leesenghua@hotmail.com; 6 Faculty of Wood Sciences and Technology, Technical University in Zvolen, 96001 Zvolen, Slovakia; kristak@tuzvo.sk; 7 Department of Agro-Industrial Technology, Universitas Padjadjaran, Bandung 45363, Indonesia; 8 Department of Forest Products Technology, Faculty of Forestry, Universitas Sumatera Utara, Kwala Bekala Campus, Medan 20355, Indonesia; apri@usu.ac.id

**Keywords:** bio-polyurethane, mechanical properties, natural fiber, non-isocyanate, thermal properties

## Abstract

The textile industries need an alternative to cotton since its supply is unable to keep up with the growing global demand. The ramie (*Boehmeria nivea* (L.) Gaudich) fiber has a lot of potential as a renewable raw material but has low fire-resistance, which should be improved. In this work, the objectives were to investigate the characteristics of lignin derived from black liquor of kraft pulping, as well as the properties of the developed lignin-based non-isocyanate-polyurethane (L-NIPU), and to analyze ramie fiber before and after impregnation with L-NIPU. Two different formulations of L-NIPU were impregnated into ramie fiber for 30, 60, and 90 min at 25 × 2 °C under 50 kPa. The calculation of the Weight Percent Gain (WPG), Fourier Transform Infrared Spectrometer (FTIR), Rotational Rheometer, Dynamic Mechanical Analyzer (DMA), Pyrolysis Gas Chromatography Mass Spectrometer (Py–GCMS), Universal Testing Machine (UTM), and hydrolysis test were used to evaluate the properties of ramie fibers. The result showed that ramie fiber impregnated with L-NIPU produced higher mechanical property values and WPG than non-impregnated ramie fiber. There is a tendency that the longer impregnation time results in better WPG values, FTIR intensity of the urethane group, thermomechanical properties, crystallinity, and mechanical properties of ramie fiber. However, the use of DMC and HMT cannot replace the role of isocyanates in the synthesis of L-NIPU because it produces lower heat resistance than ramie impregnated using pMDI. Based on the results obtained, the impregnation of ramie fiber with L-NIPU represents a promising approach to increase its wider industrial application as a functional material.

## 1. Introduction

Textiles play an important role in our lives. Textiles have different mechanical and chemical properties depending on their origin [1]. The continuous decline in environmental conditions due to the impact of waste synthetic materials and the depletion of natural resources have provoked an increased scientific and public interest in the development of alternative bio-based materials from renewable sources. Natural fibers, in particular, are fully biodegradable and non-toxic materials that have no negative environmental impact [2]. The textile industry is one of the highest-polluting industries worldwide, so comprehensive research on alternative materials to replace cotton in textiles is of crucial importance [3].

The most commonly used natural fiber in textile industries today is cotton fiber. The demand for cotton fiber in Indonesia is higher than its supply due to the continued increase in demand for textiles. Indonesia can only meet 0.1% of the total need for cotton fiber, which causes the import value of cotton to continue to increase. Indonesia exports natural fiber, with an average of USD 0.841 billion per year, while its import value reached USD 2.448 billion per year from 2014–2018, which was a period that was dominated by cotton fiber, with a percentage reaching 92% [4]. One of the most important alternative natural fibers to replace cotton is ramie (*Boehmeria nivea* (L.) Gaudich) fiber. Ramie fiber, which is extracted from the plant bast, is an important raw material for the textile industry and is characterized by a lower carbon footprint than cotton. Compared to cotton, ramie has lower water consumption and also has potential as a source of nutritional animal feedstock [5]. The adoption of ramie also enables a reduction in cost associated with agricultural activities by 77.63% when compared to cotton for the medium total agricultural activity cost and medium yield estimates [3]. In comparison to glass fibers, natural fibers like ramie fiber have many distinctive advantages, such as biodegradability, good mechanical properties, low density, and higher specific stiffness. The Young’s Modulus and specific stiffness of ramie fibers are high [6]. However, ramie fiber has certain disadvantages, such as high flammability, hydrophilic nature, limited maximum temperature, and low resistance to humidity [7]. Anti-flame agents have been widely used in the textile industry. Commercially available flame retardants are currently made from a brominated reaction, such as hexabromo–cyclododecane (HBCDD), polybrominated diphenylether (PBDE), and other synthetic materials [8]. Based on that, modifying ramie fiber with natural ingredients is required to boost its heat resistance.

**Figure 1:**
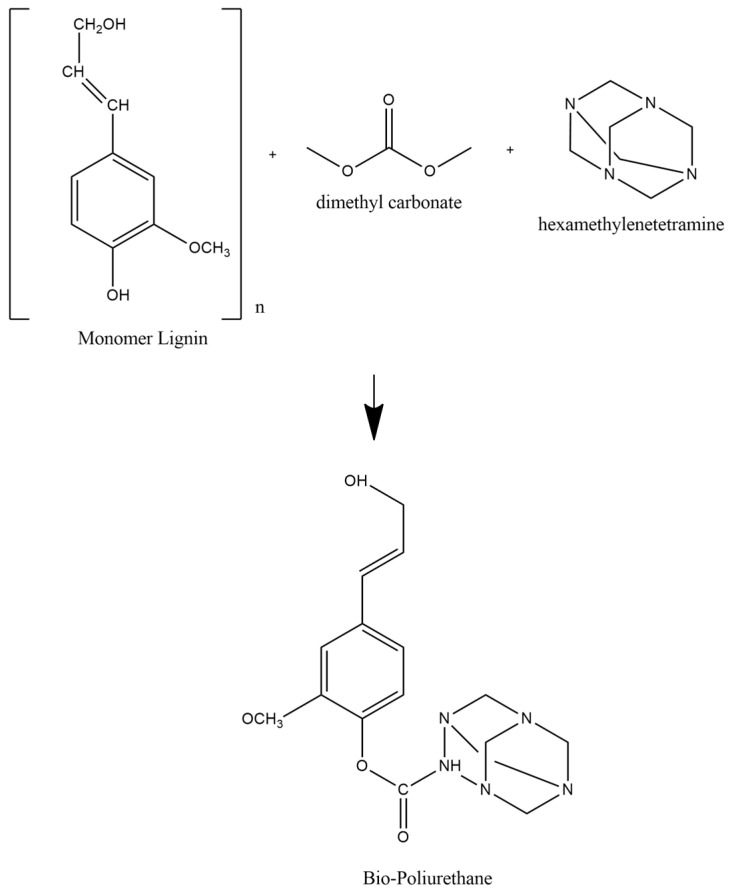
Bio-polyurethane synthesis-reaction scheme.

As industrial development took over the world economy, safety has become an important issue. A study of development in fiber, fabric, and protective clothing resulted in the growing segment of the textile industry. Fire-retardant fabrics are widely needed in various industries, such as firefighting, the chemical industry, non-clothing fabrics, and mining [9]. Modifications to increase the fire resistance of ramie fiber can be conducted by impregnation using lignin-based non-isocyanate bio-polyurethane. Polyurethanes (PUs) are a type of polymer that is distinct from most other types of plastics. Polyurethanes can be found in a variety of applications, including paints, liquid coatings, elastomers, and insulators. Polyurethanes are widely used in a variety of industries due to their high mechanical strength and biocompatibility [10]. Commercial PUs are synthesized by polycondensation reactions between isocyanates and polyols. The hydroxyl group (-OH) of polyol and the isocyanate group (-NCO) of diisocyanate combine to create PU, which is subsequently cross-linked into a urethane group with a three-dimensional network [11]. The cellulose from ramie fibers were covalently attached on the surface to exploit this chemistry reactivity, which could have applications in surface-based polymer interaction biosensors [12]. However, using isocyanates has negative environmental and human health consequences and is not sustainable. As a result, it is necessary to develop PU derived from environmentally friendly resources that do not contain isocyanates. The production of long-lasting PU necessitates the substitution of polyols with biopolyols, one of which contains lignin [10]. A schematic representation of the chemical reaction involved to form urethane bonds is presented in Figure 1.

Lignin, the second-most abundant biopolymer on earth, represents a sustainable and renewable natural feedstock for a variety of value-added applications. Plant cell walls contain 15%-to-30% lignin, which is frequently regarded as one of the most promising biomaterials due to its numerous advantages, high functionality, and renewability [13]. Various high-performance polymers, such as PU, epoxy resins, phenol–formaldehyde, and polyesters that have been synthesized with lignin as a macro-monomer, exhibit high thermal properties and mechanical strength. Lignin can also be added into other polymer matrices as a reinforcing filler to enhance the performance of composites for specific applications due to its excellent thermal stability, appealing intrinsic stiffness, antioxidant characteristics, and biodegradability. A high-value-added polymer compound made of lignin has also been employed extensively, particularly PU, which exhibits great performance and may be used in daily life [14]. Therefore, the aim of this research work was to investigate and evaluate the properties of lignin obtained from black liquor of kraft pulping, as well as the properties of the developed lignin-based non-isocyanate-polyurethane (L-NIPU), and to analyze the ramie fiber before and after impregnation with NIPU. This research has potential in industrial applications and future perspectives, such as designing more durable composites using plant fibers as fillers [15], producing road materials [16], and developing functional textile products [17].

## 2. Materials and Methods

### 2.1. Materials

Black liquor and ramie fiber as byproducts of the pulp mill’s kraft-pulping process were the primary raw materials employed in this study. The black liquor resulting from the kraft process of *Acacia mangium* wood pulp was supplied by PT Tanjung Enim Lestari Muara Enim Pulp and Paper, South Sumatra, Indonesia. Ramie fiber was obtained from CV Rabersa, Wonosobo, Central Java, Indonesia. The ramie fiber used was processed and degummed. Lignin was obtained from the isolation of black liquor from the kraft-pulping process. Lignin was standardized using Sigma–Aldrich (CAS No. 80068-05-1). In addition, other materials used were 1 M hydrochloric acid (HCl, analytical grade, Merck, Dramstadt, Germany), oxidizing agents, acetone, 20% NaOH, hexamethylenetetramine (HMT, Sigma–Aldrich, Hamburg, Germany), Dimethyl carbonate (DMC, Sigma-Aldrich, Hamburg, Germany), and distilled water.

The tools used were plastic jars, glass rods, measuring cups, Erlenmeyer flasks, test tubes, Buchner funnels, IG3 IWAKI/PYREX filter cups, freezers, filter paper, aluminum foil, petri dishes, pipettes, 5-mL volumetric pipettes, Mohr pipette 10 mL, red bulb, Duran bottle, vials, oven, analytical balance, desiccator, pH meter, mortar, magnetic stirrer, 1-L vacuum chamber (VC0918SS, VacuumChambers.eu, Białystok, Poland), UV–Vis Spectrophotometer (UV–1800, Shimadzu, Kyoto, Japan), FTIR (SpectrumTwo, Perkin Elmer, Waltham, MA, USA), dynamic-mechanical analysis (DMA), TGA4000 (Perkin Elmer, Waltham, MA, USA), Py–GCMS (Shimadzu, Kyoto, Japan), and UTM (5kN, Shimadzu, Kyoto, Japan).

### 2.2. Methods

#### 2.2.1. Lignin Extraction

In a plastic jar with 200 g of black liquor and 2000 mL of distilled water, 1 M HCl was added while being stirred until a pH meter was used to determine pH 2. The solution was stored for 24 h at room temperature (25 °C) so that the filtrate and residue were separated. The filtrate was decanted by means of a pipette, then the solution was washed with 0.7 volume of initial distilled water and stored again for 24 h at room temperature. Three separate decantation procedures were used. The residual sediment is then placed in the freezer for 24 h. The precipitate was filtered through a Buchner funnel, and the precipitate on filter paper was stored in an oven for 24 h at 45 °C. The precipitate was next ground up in a mortar and screened through a 60-mesh screen.

#### 2.2.2. Lignin Characterization

##### Moisture Content of Lignin

The petri dish spent four hours in a 105 °C oven and stored in a desiccator for 30 min. Empty petri dishes were then weighed, and the initial weight was recorded. A petri dish containing 0.1 g of lignin was weighed and then heated to 105 °C for 24 h. Samples were stored in a brush for 30 min and weighed to a constant weight. Lignin water content was calculated using the TAPPI 1997 equation as follows [18]:

(1) $$Moisture\;content\;\left(\%\right)=\frac{B-(C-A)}{B}\times 100\%$$

where:

*A* is the empty petri dish weight (g)
*B* is the initial weight (g)
*C* is the oven dry weight of sample and petri dish (g)

##### Determination of Ash Content

The porcelain cup is heated in a furnace at 525 ± 25 °C for 30–60 min and stored in a desiccator until constant. One gram of lignin sample was weighed in a porcelain cup and placed in the furnace for 6 h (525 ± 25 °C), and the original weight of the cup was recorded. The sample was put into the desiccator and weighed. The following equation was used to determine the amount of ash content (TAPPI 2007) [19]:

(2) $$Ash\;content\;\left(\%\right)=\frac{\left(C-A\right)}{B}\times 100\%$$

where:

*A* is the empty petri dish weight (g)
*B* is the initial weight (g)
*C* is the oven-dry weight of porcelain and ash (g)

##### Determination of Lignin Purity Levels

First, 3 mL of 72% H_2_SO_4_ and 0.3 g of lignin sample were combined using a magnetic stirrer at 150 rpm for 2 h. Then, 84 mL of distilled water and 3 mL of 72% H_2_SO_4_ were combined to create a blank solution. The sample was transferred to the Duran bottle and then diluted to a concentration of 4% with the addition of 84 mL of distilled water and then heated using an autoclave at 121 °C for 1 h. The filtered solution was filtered using an IG3 filter glass, which had previously been baked in the oven for 24 h (100 °C) and the initial weight was known. The filtered precipitate was then dried for 24 h at 105 °C in an oven before being weighed. The filtrate was then placed in a test tube along with a blank solution made up of 13 dilutions. A vortex was created in the solution and a UV–Vis spectrophotometer set to a wavelength of 240 nm was used to measure its absorbance. The following equation was used to determine the levels of acid soluble lignin (ASL) and acid insoluble lignin (AIL) (TAPPI 2022) [20]:

(3) $$ASL\;\left(\%\right)=\frac{UVabs\times filtrate\;volume\times amount\;of\;dilution}{\varepsilon \times A\times cuvette\;length}\times 100\%$$

(4) $$AIL\;\left(\%\right)=AIR\;\left(\%\right)-ash\;content\;(\%)$$

(5) $$Acid\;Insoluble\;Residuue\;\left(AIR\right)\left(\%\right)=\frac{C-B}{A}\times 100\%$$

where:

*ε* is the constant absorption at a certain wavelength (L/g cm)
*A* is the sample weight without water (g)
*B* is the oven dry weight of empty IG3 (g)
*C* is the oven dry weight of IG3 and AIL (g).

##### Functional Group Analysis of Lignin Using FTIR

FTIR (Spectrum Two, PerkinElmer, Waltham, MA, USA) was used to analyze the functional groups of lignin using the Universal Attenuated Total Reflectance (UATR) technique. With wavelengths ranging from 400 to 400 cm^−1^, 16 images at a resolution of 4 cm^−1^ were used to record the average accumulation.

##### Thermal Stability Anlysis Using TGA

A TGA equipment (TGA 4000, Perkin Elmer, Waltham, MA, USA) was used to conduct the thermoradimetric analysis. A 20 mL/min flow of nitrogen was applied to heat a 20-mg sample of lignin that had been weighed into a typical ceramic cup. With a heating rate of 10 °C/min, the temperature used for heating varied between 25 and 750 °C. With the use of Pyris software (Version 11, Pyris, Waltham, MA, USA), the percentage of weight loss, the rate of weight loss, and the residue were all examined.

##### Lignin Component Analysis Using Py–GCMS

First, 500–600 g of lignin samples were placed in the SF PYIEC50F eco-cup, coated with glass wool, and then examined using Py–GCMS analysis. An SH–Rxi–5Sil MS column with a film thickness of 30 m × 0.25 mm i.d. 0.25 m, 70 eV electrons, and helium as the carrier gas was used to pyrolyze the eco-cup at 500 °C for 0.1 min. The flow rate was 0.61 mL/min in the column at a pressure of 20 kPa (15.9 mL/min). The temperature profile for GC was 50 °C maintained for 1 min, followed by a 5 °C/minute temperature increase to 280 °C and a 13 min hold at 280 °C. Retention time and mass spectral data from the 2017 NIST LIBRARY program (Adaptas Solutions, Palmer, MA, USA) were compared to identify the pyrolysis products.

#### 2.2.3. Lignin Solution Preparation

A total of 0.25 g of lignin isolated from black liquor was weighed on an analytical balance, then put into a vial. Then, 25 mL of 20% NaOH was added. The 20% NaOH-dissolved lignin solution was stirred using a magnetic stirrer for 30 min until the lignin was completely dissolved.

#### 2.2.4. Lignin-Based Bio–NIPU Synthesis

Lignin Bio–PU was prepared by reaction between the lignin solution and DMC with various ratios of lignin:DMC (1:1) at 100 ± 2 °C and stirring for 15 min. The solution was then added with hexamine (HMT) with continuous stirring at 500 rpm for 30 min. Lignin-based bio–NIPU was made into two formulas, namely lignin–DMC:HMT (1:1) for Formula 1 (F1) and lignin–DMC:HMT (1:2) for Formula 2 (F2).

#### 2.2.5. Characterization of Lignin-Based Bio–NIPU

##### Analysis of Lignin-Based Bio–NIPU Functional Group by FTIR Spectroscopy

Using the Universal Attenuated Total Reflectance (UATR) approach, functional group analysis of bio-PU lignin was performed using Fourier Transform Infrared (FTIR) Spectrum Two (Perkin Elmer, Waltham, MA, USA). With wavelengths ranging from 400 to 400 cm^−1^, the average accumulation was captured using 16 scans at a resolution of 4 cm^−1^. Then, measurements were conducted at a temperature of 25 ± 2 °C.

##### Thermal Stability Analysis Using TGA

A Thermogravimetric Analyzer (TGA) device (TGA 4000, Perkin Elmer, Waltham, MA, USA) was used to conduct the thermogravimetric analysis. A 20-mg sample of bio–PU lignin was weighed into a typical ceramic crucible and heated at 20 mL/min in a nitrogen environment. With a heating rate of 10 °C/min, the temperature used for heating ranged from 25 to 750 °C. Pyris 11 software (Version 11.1.1.0492, Pyris, Waltham, MA, USA) was used to examine the percentage weight loss, weight loss rate, and residue.

##### Thermo–Mechanical Analysis Using DMA

Samples of lignin Bio–NIPU were smeared on filter paper (CAT No. 1005-125, Whatman, UK). Dynamic-mechanical measurements of bio–PU lignin on filter paper were conducted using DMA (DMA 8000, PerkinElmer, Waltham, MA, USA) dual-cantilever mode with a constant frequency pull of 1 Hz at a temperature of 20–50 °C [21]. The viscoelastic response was expressed in terms of storage modulus (E) and resistance to pressure (tan) by heating each sample at the rates 2 °C/min. The graph of the results is displayed using Pyris 11 software (Version 11.1.1.0492, Pyris, Waltham, MA, USA).

##### Viscosity Analysis Using Rotational Rheometer

The 20-mL thick extract of lignin and samples of bio–PU lignin were placed on a rotational rheometer (RheolabQC, AntonPaar, Graz, Austria) in a specific measuring cup (C-CC27, AntonPaar, Graz, Austria). In order to calculate the average viscosity, the spindle CC No. 27 was used with a continuous shear rate of 250/s at 25 °C for 120 s. The Rhe–Compass application (Version 1.33, AntonPaar, Graz, Austria) was then used to display the calculated viscosity values.

##### Chemical Component Analysis of Lignin Bio–NIPU Using Py–GCMS

Each 500–600-µg Bio–NIPU sample was placed into the SF PYIEC50F eco-cup, coated with glass wool, and subjected to PyGCMS analysis. An SH–Rxi–5Sil MS column with a film thickness of 30 m × 0.25 mm i.d. 0.25 m, 70 eV electrons, and helium as the carrier gas was used to pyrolyze the eco-cup at 500 °C for 0.1 min. The flow rate was 0.61 mL/min in the column at a pressure of 20 kPa (15.9 mL/min). The temperature profile for GC was 50 °C maintained for 1 min, followed by a 5 °C/minute temperature increase to 280 °C and a 13 min hold at 280 °C. Retention time and mass spectral data from the 2017 NIST LIBRARY program (Adaptas Solutions, Palmer, MA, USA) were compared to identify the pyrolysis products.

#### 2.2.6. Ramie Fiber Impregnation with Lignin Based Bio–NIPU

The degummed dried ramie fiber was impregnated with Lignin Bio–NIPU. Impregnation was carried out in a 1-L vacuum chamber with a two-stage vacuum pump (VC0918SS, VacuumChambers.eu, Białystok, Poland). The initial weight of the ramie fiber was recorded before vacuum impregnation. Each sample of 3 g of ramie fiber was immersed in 30 mL of bio–PU lignin, which was then impregnated at 27 ± 2 °C under a pressure of 50 kPa for 30, 60, and 90 min. The impregnated fibers were then dried for 24 h at 25 ± 2 °C. The dry ramie fiber was weighed. By dividing the difference in flax fiber mass between, before, and after impregnation by the original flax fiber mass, weight growth (%) was obtained. After that, the impregnated ramie fibers were kept in zip-lock bags for additional testing [22].

#### 2.2.7. Ramie Fiber Characterization

##### Functional Group Analysis with FTIR Spectroscopy

The Universal Attenuated Total Reflectance (UATR) technique and FTIR (SpectrumTwo, PerkinElmer, Waltham, MA, USA) were used to analyze the functional groups of ramie fiber before and after impregnation. With wavelengths ranging from 400 to 400 cm^−1^, the average accumulation was captured using 16 scans at a resolution of 4 cm^−1^. Measurements were conducted at a temperature of 25 ± 2 °C.

##### Thermal Stability Using TGA

TGA equipment (TGA 4000, Perkin Elmer, Waltham, MA, USA) was used to perform a thermogravimetric study of ramie fibers both before and after impregnation. Ramie fiber was weighed at 20 mg and cooked in a nitrogen environment at a flow rate of 20 mL/min in a typical ceramic crucible. With a heating rate of 10 °C/min, the temperature used for heating ranges from 25 to 750 °C. Pyris 11 software (Version 11.1.1.0492, Pyris, Waltham, MA, USA) was used to examine the percentage weight loss, weight loss rate, and residue.

##### Mechanical Properties Analysis Using UTM

Ramie fiber was tested for tensile strength both before and after impregnation using UTM in accordance with ASTM D 3379–75 (ASTM 2000) standard [23]. The samples were single fibers that had been cut loose from their strand links. The length of the specimens ranged from 20 mm to 30 mm, and the length of the whole fiber was around three times that of the specimen. Test specimens were subjected to a 5000 N loading and a 1 mm/min crosshead speed. The tests were carried out at a room temperature of 25 ± 2 °C. The following equation was used to determine the Modulus of Elasticity (MOE) and tensile strength [23]:

MOE=L/CA

where:

*L* = Specimen gage length, mm (or in.),
*C* = True compliance, mm/N (or in./lbf), and
*A* = Average filament area, m^2^ (or in.^2^)

Tensile Strength=F/A

where:

*F* = Force to failure, N (or lbf), and
*A* = Average filament area, m^2^ (or in.^2^)

##### Themo–Mechanical Analysis Using DMA

Dynamic-mechanical measurements of ramie fiber before and after impregnation were conducted using DMA (DMA 8000, PerkinElmer, Waltham, MA, USA) single cantilever mode with a constant frequency pull of 1 Hz at a temperature of 25–300 °C. The viscoelastic response was expressed in terms of storage modulus (E) and resistance to stress (tan) by heating each sample at a rate of 5 °C/min. Graphic results are displayed using Pyris 11 software (Version 11.1.1.0492, Pyris, Waltham, MA, USA).

##### Crystallinity Analysis Using XRD

XRD (D/Max-2500, Rigaku Miniflex II, Tokyo, Japan) analysis was used to compare the crystallinity of ramie fiber before and after impregnation using a CuK radiation source (=0.15406 nm). Each sample’s XRD pattern was obtained by scanning at room temperature at a rate of 0.020/min at an angle between 100 and 800 degrees. Using the de-convolution method, the crystallinity of ramie fiber before and after impregnation with bio–PU lignin was determined. By adjusting the curves of the Gaussian function, the single crystal and amorphous peaks of the XRD curve for each resin were deconvoluted and used for crystallinity calculations. The results of the data analysis were displayed using OriginPro 9.0 software (OriginLab Corporation, Northampton, MA, USA).

##### Morphology and Chemical Properties Analysis Using FESEM–EDX

Field Emission Scanning Electron Microscopes (FE–SEM) (Quattro S, Thermo Fisher Scientific Inc., Waltham, MA, USA) and EDX (Ultim Max, Oxford, UK) were used to scan the ramie fiber bundles and examine the morphological and chemical characteristics of the ramie fiber before and after impregnation. Use a K1 X-ray source with 3.0 kV power at 1000 times magnification.

##### Hydrolysis Test of Impregnated Ramie Fiber

Hydrolysis tests were conducted on ramie fiber before and after impregnation with lignin-based Bio–NIPU. Soaking was conducted using distilled water at room temperature and 60 °C for 1 h each. The initial weight of ramie fiber is weighed before soaking. Soaking was done with a ratio of ramie fiber:aquades 1:100. The ramie was then placed in an oven at 103 ± 2 °C for 24 h. Analysis was carried out on changes in ramie fiber weight before and after hydrolysis, changes in pH and color of the remaining soaking solution, and analysis of changes in functional groups using FTIR.

## 3. Results

### 3.1. Black Liquor and Lignin Characterization

The black liquor in this study was obtained from the kraft-pulping process of a paper mill. The water content, solids content, and pH of the black liquor are shown in Table 1.

The water content of black liquor obtained in this study was 27.81 ± 1.11%, which is differed from [24] with a yield of 10%. Meanwhile, the solids content of the black liquor in this study was 76.79 ± 0.64%, which is in the range of 65–85%, according to research by [25]. The solids content shows that the lignin in the wood is dissolved by the cooking solution [26]. The corresponding results were also obtained at the pH of black liquor, which was 12.14 ± 0.03, and the results were in line with [27], which is in the range 12–13. This indicates that the black liquor is alkaline as the solution used in the kraft pulp cooking process was alkaline, which is NaOH and Na_2_S. In addition to these parameters, ramie fiber also contains lignocellulosic polymers, which include cellulose (72.0%), hemicellulose (5.0–16.7%), lignin (0.6–0.8%), pectin (2.0%), wax (0.3%), ash, and moisture (negligible content) [28]. This study used lignin isolated from black liquor. The yield of lignin (Table 2) was slightly higher than that of [29].

The lignin obtained had a water content and ash content of 5.07 ± 0.71% and 0.31 ± 0.19%, respectively. This result is different from the findings reported by [30] and [31], who reported a moisture content of 8.05% and an ash content of 8.25%. The high-water content was due to the presence of impurities containing hydrophilic compounds. Meanwhile, the ash content indicated impurities in lignin in the form of minerals such as sodium (Na) and sulfur (S) from chemicals in the kraft cooking process, namely NaOH and Na_2_S [30,31]. This shows that the lignin in this study was better because it contained fewer impurities.

The percentage of total lignin is the sum of the Acid Insoluble Lignin (AIL) and Acid Soluble Lignin (ASL). The lignin AIL content of black liquor was 82.54 ± 0.96%, while the ASL content was 12.77 ± 0.67% (Table 2). Based on these data, the percentage of lignin purity obtained was 95.32 ± 0.61%. This is a higher percentage than the published work, which was only 84.21% [32]. Differences in lignin content can cause differences in the content of guaiacyl, methoxyl, and syringyl monomers in lignin. In addition, lignin is a strong component because its characteristics are very stable against heat treatment to temperatures above 200 °C. Lignin-based flame retardants have been employed to produce high-performance and sustainable polymeric materials. The proclivity of lignin to produce high-char residues during thermal decomposition improves the flame retardancy of the final polymer composites by operating in the condensed phase [33].

#### 3.1.1. Functional Groups of Lignin

**Figure 2:**
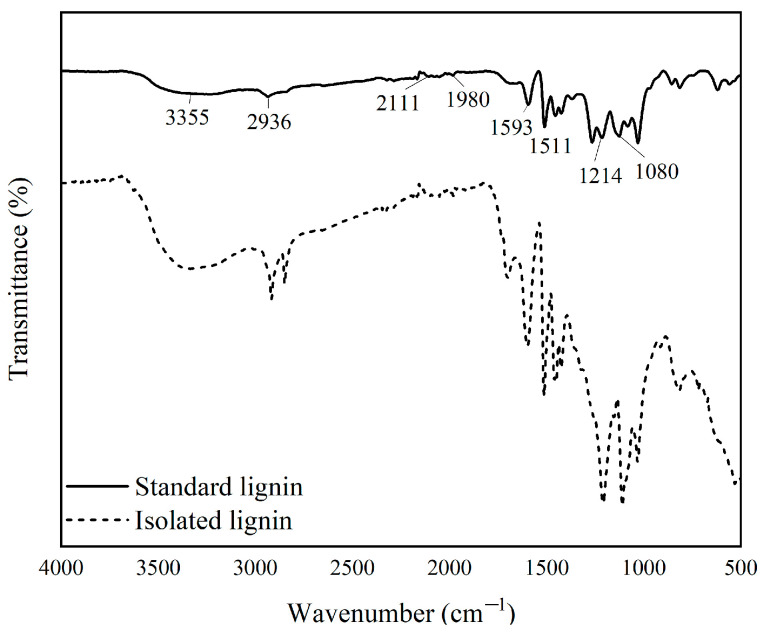
FTIR spectrum of L-isolated and L-standard.

Black liquor’s standard lignin (L-Standard) and isolated lignin (L-Isolation) underwent functional group analysis utilizing FTIR (Figure 2).

This analysis was conducted to detect complexes and electrostatic interactions formed in the test sample. Based on the resulting spectrum, it was determined that the absorption bands on L-Isolation and L-Standard had relatively the same results but with different transmittance intensities at certain wave numbers. This shows that the lignin isolation did not damage the lignin structure. Broad wave absorption occurred in the absorption band 3550–3200 cm^−1^, with peaks at wave numbers 3355 cm^−1^ on L-Standard and 3347 cm^−1^ on L-Isolation. The peaks at this wave number indicated the presence of aliphatic OH and phenolic OH groups. The spectrum showing the C–H group associated with the CH_2_ and CH_3_ groups was seen in wave numbers 2936–2917 cm^−1^; the peaks that appeared in wave numbers 1710–1698 cm^−1^ indicated the presence of C=O groups; aromatic skeletal vibrations were present in wave number 1601–1593 cm^−1^; and the C–C group of the aromatic ring was observed at wave numbers 1514–1511 cm^−1^. The wave number 1515–1505 cm^−1^ described the aromatic ring of the phenylpropane skeleton [31].

The peaks that appeared at wave numbers 1470–1460 cm^−1^ were equally found in L-Standard and L-Isolation. This demonstrates that lignin’s aromatic structure did not alter during the separation procedure. In the area 1210–1220 cm^−1^ and 1030 cm^−1^, there were phenolic OH groups: C–O ether groups in syringyl and guaiacyl and C–H groups in guaiacyl. The wave numbers 1269 cm^−1^ and 1085–1030 cm^−1^ indicated the presence of aliphatic O–H and ether groups. The peaks that appeared at these wave numbers had a greater intensity in L-Standard than in L-Isolation. This is because standard lignin uses needle leaf wood, which has a higher amount of guaiacyl lignin, while isolated lignin was obtained from black liquor from processed mangium wood, which is a broadleaf species [34].

#### 3.1.2. Thermal Stability Analysis

The TGA–DTG thermogram analysis depicted in Figure S1 was used for evaluating the thermal stability of lignins. Under a nitrogen atmosphere, the TGA–DTG thermogram analysis was conducted to demonstrate the relationship between mass change or lignin degradation with a particular temperature rise. Standard lignin was used as a comparison in this analysis. Based on TGA–DTG research, three stages of lignin breakdowns were determined. The lignin lost weight during the first step at a temperature of 25 to 75 °C as a result of water evaporation. At this point, there was a 0.2–1% weight decrease. The next step involved weight loss due to carbohydrate decomposition, which occurred between 75 and 370 °C. Lignin lost up to 10% in weight at this stage, at 235 °C in isolated lignin and 259 °C in standard lignin (Table 3) with a weight loss rate of 0.5%/°C, then it lost weight by 25% at room temperature, i.e., 247 °C in insulating lignin and 259 °C in standard lignin.

The next stage, namely lignin degradation, occurred with the widest range, namely at a temperature from 360 °C to 750 °C. At this stage, the lignin lost weight up to 50% at 597 °C in standard lignin, while isolated lignin was at 644 °C. The release of CO, CO_2_, and H_2_O chains from the lignin structure and the breakdown of low-+-molecular weight lignin polymers were both connected to this process [35]. The DTG curve is a weight derivative of the lignin weight loss value. At its highest peak, lignin degradation reached its maximum range at a temperature of 370 °C, with an average weight loss of 2.5%/°C.

The residue level produced on standard lignin was 42.41%, while isolated lignin produced a higher residue of 46.25%. The residue is the mass that is not degraded during the heating process in the TGA test. The yield of lignin residue was higher than tannin, which reached 19.43% [36]. This indicates that lignin had greater heat stability than tannins. Lignin has better heat-resistant qualities since it comprises a variety of aromatic groups that are typically destroyed at high temperatures [37].

#### 3.1.3. Py–GCMS of Lignin

The composition of the lignin present in L-Standard and L-Isolation was analyzed by Pyrolysis gas chromatography mass spectrometry (Py–GCMS). Thermal fragmentation at high temperatures up to 500 °C and in an oxygen-free environment was used to perform pyrolysis. Although they demonstrated varying intensities, the commatograms generated on L-Standard and L-Isolation were essentially identical (Figure S2).

The analysis’s findings demonstrated that during a retention duration of 21–32.5 min, isolated lignin produced more siringil than guaiacyl and hydroxyphenyl. Meanwhile, standard lignin produced more guaiacyl at a retention time of 12.5–28.5 min. This result is in line with the results of lignin FTIR analysis, which showed that the standard lignin was guaiacyl lignin from needle leaf wood, while the isolated lignin was siringil–guaiacyl lignin from broadleaf wood. Based on the research results, the S/G ratio of L-Isolation was 0.96 with a S/G/H ratio of only 0.045. The main peaks on the chromatogram were Guaiacol (G1), Syringol (S1), Guaiacol 4-methyl (G2), Syringol-4methyl (S2), and Guaiacol 4-vinyl (G5). An increase in the abundance of H-units in lignin will cause a decrease in S-units and G-units, which will cause a decrease in S-units and G-units due to the loss of the methoxyl group (-OCH3) from S-units and G-units due to thermal degradation [38]. The S/G ratio contributes to the chemical reactivity of various technical lignines. The S/G ratio is influenced by the method, bleaching process, and bioethanol production during pulping [39]. The results of the S/G/H ratio are used as a reference in the manufacture of lignin-based products.

### 3.2. Lignin Bio–NIPU Characteristics

#### 3.2.1. Functional Group Analysis

**Figure 3:**
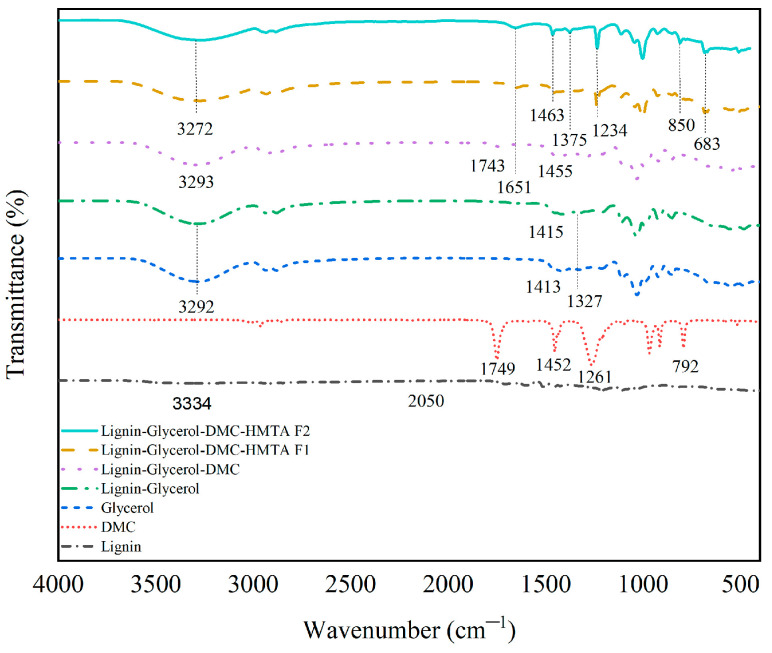
FTIR spectra of lignin Bio–NIPU.

Confirmation of the results of the synthesis of lignin-based Bio–NIPU was conducted by functional group analysis using FTIR. The functional group spectrum of lignin-based Bio–NIPU is shown in Figure 3. Analysis was carried out at each stage of the synthesis process of lignin-based Bio–NIPU to observe changes in functional groups at each manufacturing stage. Basedon the data obtained, there was a broad peak at wave numbers 3400–3200 cm^−1^, which appeared after the addition of glycerol. This indicated the formation of N–H groups in the primary aliphatic structure of the amine. The urethane bond is formed from the reaction between the OH groups of lignin and the N=C=O group [40]. In addition, there was a peak shift at wave numbers 1743 cm^−1^ to 1651 cm^−1^ from lignin–glycerol–DMC after being converted into lignin-based Bio–NIPU in both Formula 1 and Formula 2. The development of a new peak at wave number 1234 cm^−1^ indicated the production of C–N groups as a marker for the formation of urethane, and this signifies the formation of the C=O group from the bond urethane. The existence of an absorption band at wave number 850 cm^−1^, which is a bending vibration of the methyl group, further supported this [41]. Based on this, it was determined that black liquor’s lignin can be utilized as a polyol to create Bio–NIPU.

#### 3.2.2. Lignin Bio–NIPU Component

As part of an examination to ascertain the composition contained in lignin, commercial pollutants, lignin-based Bio–NIPU (L-NIPU) F1 and F2, pyrolysis gas chromatography, or mass spectrometry (Py–GCMS) was conducted (Figure S3). Thermal fragmentation at high temperatures up to 500 °C and in an oxygen-free environment was used to perform pyrolysis. Based on the GCMS analysis, at a retention time of 1.95 min, lignin showed a relatively high CO_2_ component, as well as F1, F2 Bio–NIPU, and a little in commercial polyurethane. In Bio–NIPU, it is known that there are components with relatively high concentrations such as methylamine and N,N-dimethyl (12.9%) at a retention time of 2.072 min originating from hexamine and DMC, which are used in the synthesis of Bio–NIPU. In addition, there were also compounds such as methyamine, propenol, and methanamine in the two Bio–NIPU formulas with different relative concentrations. Whereas in commercial polyurethane, there are characteristic compounds derived from PMDI, namely Diehylexyl phthalate and ethanol,2-butoxy.

#### 3.2.3. Lignin Bio–NIPU Viscosity

In this study, an impregnation agent composed of lignin-based Bio–NIPU resin was used to enhance the properties of ramie fiber. Based on this, the resulting lignin-based Bio–NIPU is expected to have a low viscosity value so that it can easily penetrate to the ramie fiber. Viscosity is the thickness of a fluid. The more viscous a fluid is, the harder it is for it to flow and, consequently, the harder it is for an object to move through the fluid. The impact of the impregnant infiltrating the pores is greater with lower viscosity [42]. Furthermore, the cohesion strength value must be understood in order to determine the resin’s internal strength, which will influence how the materials to be bonded interact. Knowing the cohesive strength of the resin is crucial because internal resin damage will weaken the bond between the fiber and resin [43]. A solution’s viscosity value will have an impact on the impregnation activity. As the viscosity value will be related to the cohesion strength value, excessive viscosity will cause the resin to only perfectly adhere to the surface of the impregnant material.

Graphical representation of the results obtained for the viscosity, cohesion strength, and relaxation modulus of the two lignin-based Bio–NIPU formulas is presented in Figure S4. It was determined that the average values of viscosity, cohesion strength, and relaxation modulus of Bio–NIPU F1 (283.47 mPa·s; 70.87 Pa; and 0.0097 Pa) were higher than those of Bio–NIPU F2 (235.05 mPa·s; 58.76 Pa; and 0.0080 Pa). The difference in viscosity in the two formulas is thought to be due to the addition of more hexamine solution to F2, resulting in a lower viscosity value because it contains more water. As the NCO/OH ratio rises, more urethane linkages will be produced to create hard segments in bio-polyurethane, increasing the viscosity that results from the interaction of hydrogen bonds between isocyanate and polyol molecules [44], while the cohesion strength is influenced by the interaction and molecular strength contained in the resin. The relaxation modulus of the two Bio–NIPU formulas had a relatively similar pattern. The relaxation modulus of the two formulas decreased at the beginning of the test time and then decreased after 40 s of testing before becoming constant. This shows that both resins had time-dependent characteristics, which can also be seen in the viscosity and cohesive strength decreasing constantly with increasing time.

#### 3.2.4. Thermal Stability Analysis

**Figure 4:**
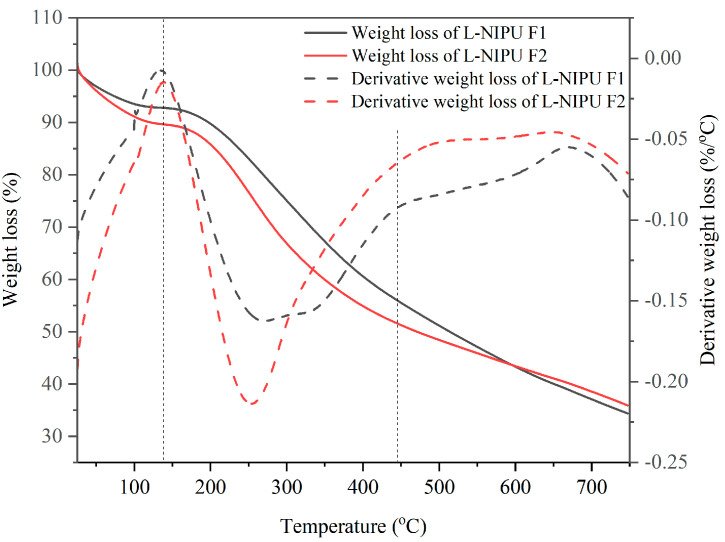
Thermal stability L–NIPU using TGA and DTG.

Thermal stability analysis was carried out using TGA. This analysis is used to determine the boiling point of esters and monitor transesterification reactions [45]. The resulting thermal-stability thermograms for F1 and F2 Bio–NIPU based on lignin were different (Figure 4).

Both formulas at first lost weight at 125 °C as a result of water evaporation and chemical degradation as a result of weak links formed during the polymerization process [46]. The influence of the resin’s urethane bond breakdown process led to the weight loss that occurred at 450 °C, with a drop in resin weight that ranged from 0.7%/°C in F2 to 0.8%/°C in F1. Both resins decreased by 10% at 196 °C on F1 and at 116 °C on F2, then they decreased by 25% at 299 °C on F1 and 257 °C on F2, and decreased by 50% at 513 °C on F1 and 467 °C on F2. Even though F1 experienced a slower weight loss than F1 in the heating process, it occurred at the end of heating (750 °C) (Table 4).

Lignin-Bio–NIPU F1 resin produces less residue (34.43%) compared to F2 (35.92%). This result is smaller than that of resin made with isocyanate (Bio–PU), which produces a higher residue (56.54%) [47]. This shows that the use of DMC and HMT in the synthesis of Bio–NIPU cannot replace the role of isocyanates in terms of increasing thermal stability. The use of isocyanate or pMDI can increase thermal stability because it is a type of thermosetting resin [48].

#### 3.2.5. Analysis of Viscoelastic and Thermochemical Properties

Dynamic Mechanical Analysis (DMA) was used to conduct an analysis to investigate a polymer’s viscoelasticity and thermomechanical characteristics in relation to its molecular structure. DMA testing was conducted on F1 and F2 of lignin-based Bio–NIPU. The thermomechanical properties are displayed through three parameters, namely storage modulus (E′), loss modulus (E″), and tan delta (tan δ), which describe the surface bonding of the composite (Figure S5). The temperature increase and the glass transition temperature (Tg) produced by the tan delta peak determine the storage modulus and loss modulus. The pattern produced by the storage modulus curve is directly proportional to the resulting loss modulus curve. This increase is thought to be caused by interactions between the chemicals used. Meanwhile, the tan delta value is inversely proportional to the storage modulus and loss modulus.

**Figure 5:**
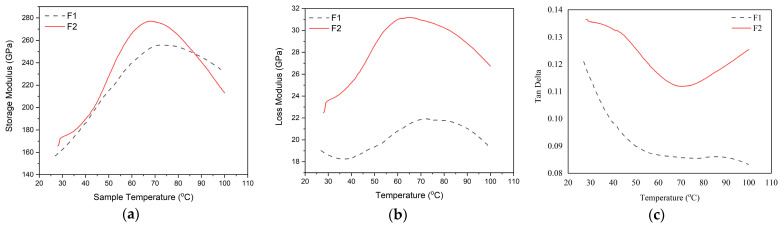
(**a**) Storage modulus; (**b**) Loss modulus; and (**c**) Tan delta of Lignin Bio–NIPU.

Viscoelastic and thermochemical tests were conducted by heating each sample at a heating rate of 2 °C/min. The storage modulus value of F1-based lignin-based Bio–NIPU (156.89 GPa) was lower than F2-based lignin–NIPU (165.71 GPa) at an initial heating temperature of 25 °C (Figure 5a).

The value of storage modulus F1 then increased until it reached the highest peak at 255.89 GPa at a temperature of 72.5 °C. In F2, it also experienced an increase to a temperature of 68.06 °C, with the highest peak at 277 GPa. The increase in the value of the storage modulus was due to an increase in the stiffness of the material, which will affect the elastic response and the material’s ability to retain the energy provided. The higher the storage modulus value, this indicates a hardening process in the sample that occurs due to polymerization that occurs during the synthesis process of lignin-based Bio–NIPU, where the highest storage modulus value was produced in F2 lignin-based Bio–NIPU. Factors that affect the high storage modulus value are the bonds that are formed more tightly and stiffly so that the material is more resistant to the movement of molecular chains [49].

Loss modulus (E″), which relates to the internal energy loss brought on by flexible deformation, internal friction, relative molecular motion, relaxation processes, phase transitions, and morphological changes, is a measure of a material’s viscosity response [50]. The E″ values of Bio–NIPU F1 and F2 tended to have the same pattern increased after the temperature was ±40 °C until it reached the highest value at 76.8 °C on F1 (21.81 GPa) and 65.53 °C on F2 (31.17 GPa) (Figure 5b). The highest E″ value was produced by Bio–NIPU based on lignin F2, which was also obtained at the previous storage modulus value. The energy lost during viscous deformation, which cannot be changed when a material changes shape under specific pressure and temperature, can also be used to determine the material’s viscosity and is represented by the resulting E″ value. To assess the material’s low temperature qualities, the glass transition temperature can be employed, which is the temperature that corresponds to the peak point of this loss modulus curve.

The ratio of storage modulus and loss modulus is called tan delta. Figure 5c shows the tan delta value of Bio–NIPU based on lignin F1 and F2. At the initial to final temperature, the tan delta value of F2 was higher than that of F1 (Figure 5c). The tan delta value of Bio–NIPU F1 continued to decrease from an initial temperature of 25 °C (0.12) to a final temperature of 100 °C (0.08), while the value of tan delta F2 decreased from an initial temperature of 25 °C (0.13) to reach the lowest peak at 70.01 °C (0.11) and then increased to a final temperature of 100 °C (0.12). The proportional loss modulus and the material’s capacity for irreversible deformation increased with increasing tan values. The reason for this larger value is that there was a greater contact between the DMC and HMT materials and the hydroxyl groups (polyphenols) created by tannins, which led to a polymerization reaction and an increase in the ratio of E′ to E″. This means that less deformation energy is dissipated as heat. The increase in the tan δ value of the resin was due to internal friction and viscoelastic energy dissipation, which increased with increasing temperature; this shows that the stiffer polymer molecule resulted in a decrease in molecular movement so that the interfacial bonding of the polymer chain F2 was better than F1.

### 3.3. Ramie Fiber Characterization

#### 3.3.1. Weight Percentage Gain (WPG)

Confirmation of the success of the lignin-based Bio–NIPU impregnation process can be seen from the percentage increase in ramie fiber weight after impregnation. Ramie fiber was impregnated using Bio–NIPU based on lignin F1 and F2, with three variations of impregnation time, namely 30, 60, and 90 min. Impregnation using both lignin-based Bio–NIPU formulas with three variations of length of time resulted in different WPG values (Table 5).

It is known that F2-impregnated ramie provided a marginally higher value than F1 based on the WPG data. However, the treatment with the lignin-based Bio–NIPU formula was not substantially different from the WPG value of ramie fiber after impregnation, according to the findings of the analysis of variance with a 95% confidence interval. This is because the viscosity between the two formulas was still in the same range, namely 200–300 mPa·s. According to [42], the low viscosity of the impregnation material will ensure it is easier for the material to enter the pores of a material. Increasing the amount of impregnation material that manages to get into the pores of the material will cause the porosity of the material to decrease because the pores are properly filled. The impregnation action penetrates into the material’s pores more effectively the lower the viscosity value of the impregnated material is.

Variations in the length of impregnation time were conducted for the two lignin-based Bio–NIPU formulas. The extended treatment period of impregnation had a significant impact on the WPG value of ramie fiber after impregnation, according to the findings of the analysis of variance with a 95% confidence interval. This resulting WPG values increased with the increase of impregnation time (Table 5). The impregnation time of 90 min resulted in the highest WPG value for ramie fibers impregnated with both F1 lignin-based Bio–NIPU and F2 lignin-based Bio–NIPU. The highest WPG value was obtained for Bio–NIPU F2-impregnated ramie fiber with a percentage gain of 51.30 ± 2.52%, followed by F2 lignin-based Bio–NIPU-impregnated ramie fiber with a WPG value of 45.05 ± 3.97%.

#### 3.3.2. Functional Groups of Impregnated Ramie Fiber

**Figure 6:**
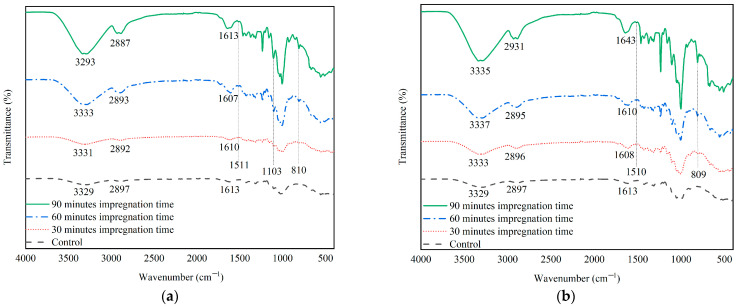
Functional group analysis ramie fibers after impregnation (**a**) Lignin-Bio–NIPU–F1; and (**b**) Lignin-Bio–NIPU–F2.

Changes in ramie fiber functional groups after impregnation were observed using the Fourier Transform Infra Red (FTIR) instrument. Analysis was performed to confirm the presence of urethane groups after impregnation using lignin-based Bio–NIPU. Based on the analysis performed, the absorption bands obtained that tended to be the same for each treatment (Bio–NIPU formula and impregnation time) but had different intensities (Figure 6a,b).

Both formulas produced the same two absorption patterns, where the longer the impregnation time, the clearer the visible peak intensity. There was a broad peak at wave numbers 3333–3293 cm^−1^ in F1-impregnated ramie fiber and 3337–3329 cm^−1^ in F2-impregnated ramie fiber, which indicated the presence of –OH groups in the same ramie fiber as non-impregnated ramie fiber with higher impregnated fiber intensity tall. Furthermore, there was a shift in wave numbers from 2897 cm^−1^ to 2887 cm^−1^ in F1-impregnated ramie fiber and from 2897 cm^−1^ to 2931 cm^−1^ in F2-impregnated ramie fiber, which indicated the presence of N–H groups in the ramie fiber after impregnation with the Bio–NIPU lignin base, which was also found in lignin-based Bio–NIPU (Figure 4). Then, a peak was formed at wave number 1600 cm^−1^, indicating the formation of C=O urethane from Bio–PU. The wave numbers of 1511 cm^−1^ in F1-impregnated ramie fiber and 1510 cm^−1^ in F2-impregnated ramie fiber indicated the activity of C–N groups originating from primary and secondary amides. Furthermore, wave number 1103 cm^−1^ shows C=O vibrations of Bio–PU and C–O–C ether bonds, respectively. This was also confirmed by the presence of absorption bands that appeared at wave numbers 810 cm^−1^ in the F1-impregnated ramie fiber and 809 cm-1 in the F2-impregnated ramie fiber, which is a bending vibration of the methyl group [41]. The bonds formed in lignin-based Bio–NIPU and impregnated Ramie fiber illustrate that lignin-based Bio–NIPU can enter and seep into the ramie fiber.

#### 3.3.3. Thermal Stability Analysis of Ramie Fiber

**Figure 7:**
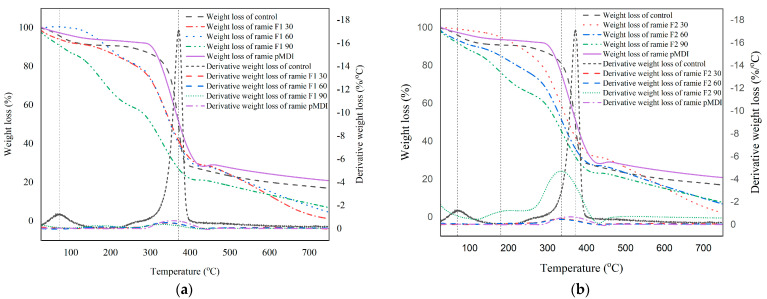
Thermal stability analysis using TGA. (**a**) Ramie fiber impregnated with lignin-based Bio–NIPU F1; (**b**) Ramie fiber impregnated with lignin-based Bio–NIPU F2.

The thermal stability of ramie fiber before and after impregnation with Bio–NIPU was analyzed using TGA. The thermograms of ramie fibers after impregnated with lignin-Bio–NIPU-impregnated differed each other (Figure 7).

The weight loss of ramie fiber at 25–100 °C occurred due to the loss of water in the fiber and the decomposition of components with low molecular weights so that the weight loss occurs below 10%. A drastic reduction in weight occurred after 300 °C for all ramie samples. The heavy derivative thermogram (DTG) showed the first peak at 60–70 °C in all ramie samples except 90 min for Bio–NIPU F2-impregnated ramie, with the peak appearing at 190–210 °C. This stage was the initial degradation of the fiber due to loss of water, with a peak indicating a weight loss of 2%/°C. The second peak occurred between temperatures of 370–380 °C, which indicated a degradation process of cellulose and lignin [51].

The thermogram of the TGA analysis results also shows the process of reducing the ramie fiber weight before and after impregnation, with different results (Figure 7). Weight loss in each weight occurred at different temperatures (Table 6).

The weight loss of 10% occurred in the control ramie occurred at 196 °C, and pMDI-impregnated ramie fiber occurred at a higher temperature of 303 °C, while the Bi–NIPU impregnated ramie fiber at a lower temperature in the range of 78–224 °C. The weight loss of 25% in the control ramie fiber occurred at 299 °C, while pMDI impregnated ramie fiber at 336 °C. In addition, the Bio–NIPU impregnated ramie at a lower temperature, namely in the range of 170–300 °C. However, 50% weight loss of control ramie occurred at 513 °C, greater than that of pMDI-impregnated ramie at 373 °C, and Bio–NIPU impregnated ramie at 303–353 °C. This reduction in weight occurred as a result of lignin degradation at 300–400 °C, which determines the limit of thermal stability [52]. The residue produced on pMDI-impregnated ramie fiber was higher than that of non-impregnated ramie and Bio–NIPU-impregnated ramie (Table 6). The amount of residue created increased with the length of the impregnation time. However, it was still less than the control residue in value. The Bio–NIPU formula with the use of DMC and HMT cannot replace isocyanate in the synthesis of Bio–PU. The use of isocyanate (pMDI) is better because it is a thermosetting resin with better thermal stability. Good results of the synthesis of Bio–NIPU were produced by [43], who used DMC and Hexamethylene diamine (HMDA) as substitutes for the role of isocyanates. This is presumably due to the use of HMT in this study, which has a closed aromatic ring form so it is not more reactive than HMDA with an open aromatic ring.

#### 3.3.4. Thermo-Mechanical Analysis of Ramie Fiber

**Figure 8:**
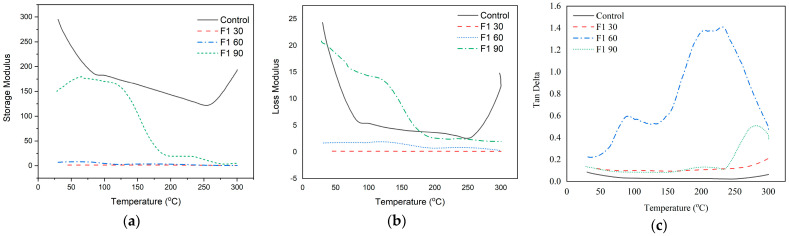
(**a**) Storage modulus; (**b**) Loss modulus; and (**c**) Tan delta of ramie fiber before and after impregnation with F1 lignin-based Bio–NIPU.

DMA testing was carried out on lignin-based F1- and F2-impregnated ramie Bio–NIPU. The thermomechanical properties are displayed through three data, namely storage modulus (E′), loss modulus (E″), and tan delta (tan δ), which describe the surface bonding of the composite. Based on the data generated, it is known that the values of Storage Modulus, Loss Modulus, and Tan Delta of F1 impregnated RAM fiber, with variations in impregnation time producing different values (Figure 8).

The storage modulus value of ramie without Bio–NIPU impregnation (control) had the highest value at an initial temperature of 25 °C (295 GPa), and then continued to decrease with an increasing temperature up to 253.2 °C (121.77 GPa). The increase in the storage modulus value was clearly seen in F1 Bio–NIPU-impregnated ramie fiber with an impregnation time of 90 min from an initial temperature of 25 °C (149 GPa), then it increased to a temperature of 66.95 °C (178 GPa) before decreasing drastically after a temperature of 130.69 °C. The E″ value of Bio–NIPU F1 impregnated ramie with an impregnation time of 30 and 60 min, which tended to have the same pattern. The E″ value of the control ramie decreased from an initial temperature of 25 °C (24.32 GPa) to reach the lowest value at 249.18 °C (2.56 GPa). The difference was seen in the F1-impregnated ramie after 90 min, where the resulting E″ value decreased slowly to 125.15 °C (13.16 GPa) but then decreased drastically to 300 °C (1.99 GPa). Meanwhile, it is known also that the value of tan delta ramie impregnated with Bio–NIPU F1 60 at 275 °C (1.46), followed by F1 90 at 265 °C (0.56).

**Figure 9:**
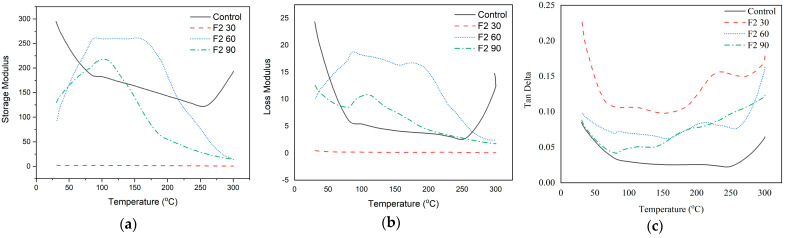
(**a**) Storage modulus; (**b**) Loss modulus; and (**c**) Tan delta of ramie fiber before and after impregnation with F2 lignin-based Bio–NIPU.

The storage modulus of F2-impregnated ramie fiber increased from 25 °C (93.67 GPa) to 91 °C (261 GPa), remained steady until 159 °C (260 GPa), and then proceeded to decline (Figure 9).

The same thing happened to F2-L-NIPU-impregnated ramie fiber with an impregnation time of 90 min starting from an initial temperature of 25 °C (129.65 GPa), then increasing to a temperature of 112 °C (212.95 GPa) until then decreasing. The peak E″ value of the highest Bio–NIPU F2-impregnated ramie was produced with an impregnation time of 60 min, followed by 90 min. The E″ value of F2 impregnated ramie 60 min at the start of heating (10.21 GPa); it increased until it reached the highest peak at 87.78 °C (18.74 GPa), decreased slowly to a temperature of 175.45 °C (16.74 GPa), and then decreased drastically. Meanwhile, 90 min F2-impregnated ramie at an initial heating temperature of 25 °C (12.55 GPa) decreased to 79.63 °C (8.52 GPa), then it increased again until it reached the highest peak at 108.99 °C (10.83 GPa) before decreasing.

The higher storage modulus value indicated a hardening process in the sample that occurred due to polymerization that occurs during the synthesis process of lignin-based Bio–NIPU, where the highest storage modulus and loss modulus values were produced in lignin-based F2 Bio–NIPU-impregnated ramie fiber with an impregnation time of 60 min, followed by F2 with an impregnation time of 90 min. Factors that affect the high0storage modulus value are the bonds that are formed more tightly and stiffly so that the material is more resistant to the movement of molecular chains [49].

#### 3.3.5. Crystallinity Analysis

Crystallinity analysis was carried out to determine the presence of a crystal structure, which was proven by using the X-ray diffraction (XRD) test. Crystallinity tests were conducted on ramie both before and after impregnation using F1 and F2 Bio–NIPU with three variations of immersion time. Based on the results of the analysis, a similar diffractogram peak was obtained for each ramie sample with different intensities (Figure S6). Based on the results of analysis on ramie fiber without impregnation, it produced peaks at a diffraction angle of 22°, which is cellulose crystallography I, and peaks at angles of 14.76° and 15.03°, which are cellulose crystallographic planes II. This shows that ramie fiber had high cellulose content [53], whereas the impregnated ramie fiber produced new peaks at a diffraction angle of 20.14°; 30.88°; and 44.15° (ramie-impregnated F1 Bio–NIPU based on lignin) and a diffraction angle of 20.28°, 30.98°, and 44.23° (F2 Bio–NIPU-impregnated ramie based on lignin), which indicated an increase in ramie fiber crystallinity after impregnation. The higher the crystallinity, the stiffer the resulting fiber will be.

**Figure 10:**
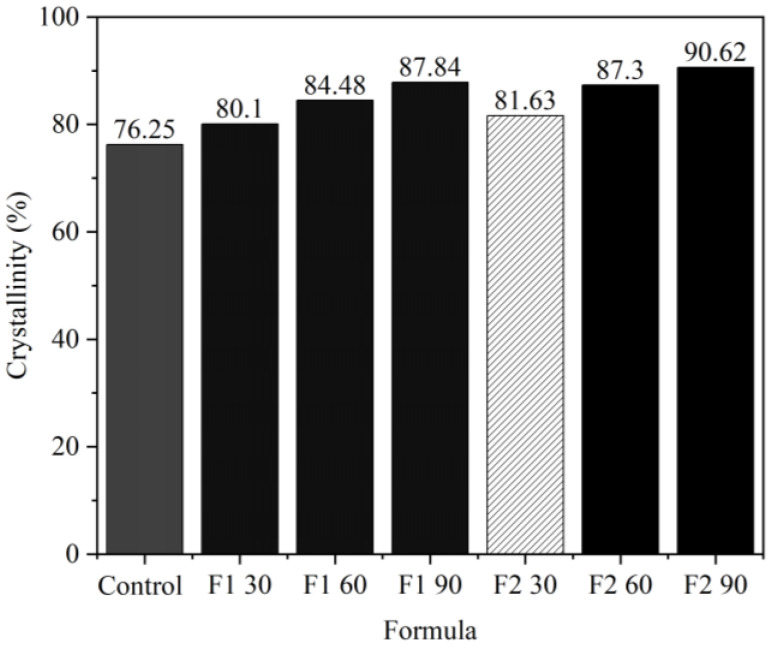
Crystallinity of ramie fiber before and after impregnation with lignin-based Bio–NIPU.

The percentage of crystallinity was calculated based on the peaks of the diffractogram obtained. Crystallinity is strongly influenced by the cellulose content of a material and greatly affects its mechanical properties. By comparing the overall area of the crystalline peaks to the combined area of crystalline and amorphous peaks, the percentage of crystallinity is determined. Using both F1 and F2 lignin-Bio-NIPU, the crystallinity value increased as the impregnation period increased. Bio–NIPU (Figure 10).

The crystalline percentage increased from untreated fiber (76.25%) to reach the highest value in F2 Bio–NIPU-impregnated ramie fiber (90.62%), followed by F1 Bio–NIPU (87.84%). Based on these results, it is known that the percentage of crystallinity is affected by the length of impregnation time. Crystallinity affects the strength of a material, which includes compressive strength, hardness, and stiffness. The mechanical strength increases with the crystallinity percentage. In addition, the increase in crystallinity causes denser intermolecular chains, which are filled with the impregnation material so that it is difficult for water to enter the impregnated material [54].

#### 3.3.6. Morphology and Chemical Analysis of Ramie Fiber

**Figure 11:**
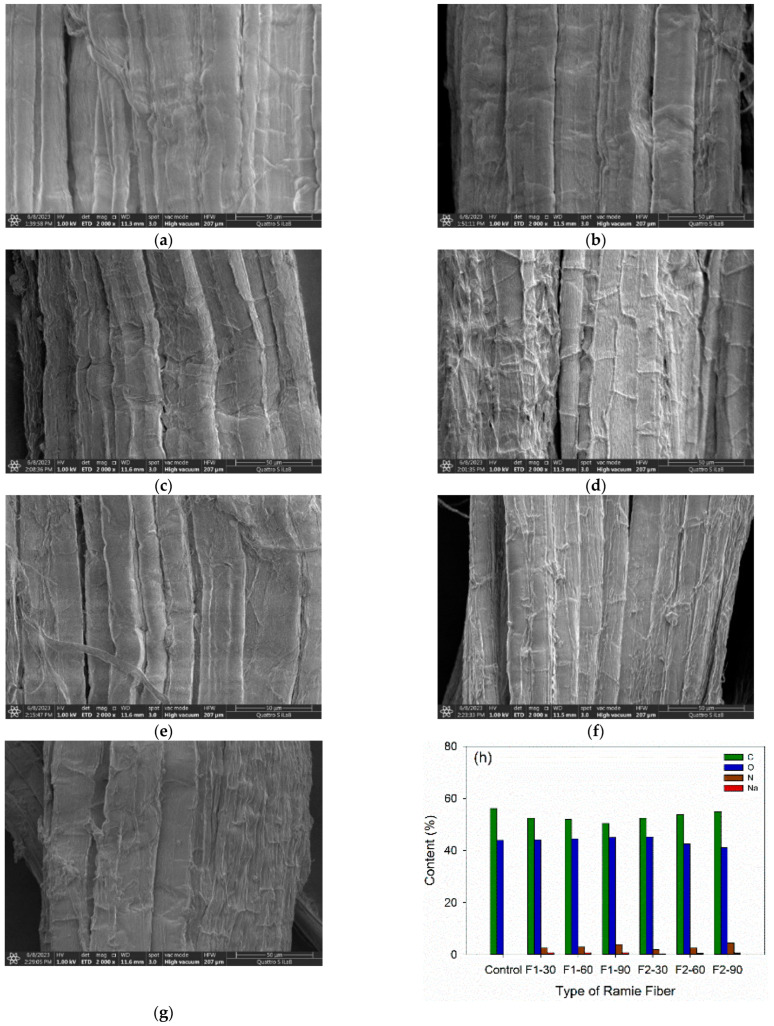
FE–SEM combined with EDX analysis of ramie fibers impregnated with lignin-based non-isocyanate polyurethane (L-NIPU) resins: (**a**) Micrograph of original ramie fibers; (**b**) Micrograph of ramie impregnated with L-NIPU–F1 resin for 30 min; (**c**) Micrograph of ramie impregnated with L-NIPU–F1 resin for 60 min, (**d**) Micrograph of ramie impregnated with L-NIPU–F1 resin for 90 min; (**e**) Micrograph of ramie impregnated with L-NIPU–F2 resin for 30 min; (**f**) Micrograph of ramie impregnated with L-NIPU–F2 resin for 60 min; (**g**) Micrograph of ramie impregnated with L-NIPU–F2 resin for 90 min; (**h**) EDX of original ramie fibers impregnated with L-NIPU resins.

Using FE–SEM EDX, it was possible to compare the surface morphology and chemical characteristics of ramie fiber before and after being impregnated with lignin-based Bio–NIPU. Ramie fiber produced fibers with better surface morphology prior to impregnation (Figure 11a) compared to ramie fiber after impregnation using lignin-based Bio–NIPU (Figure 11b–g). Based on the data collected, it can be stated that the final surface morphology will be rougher and darker with the increase of impregnation time. This is in line with the increase in ramie fiber crystallinity that occurred with increasing impregnation time. The color becomes darker because the ramie fiber bonded with lignin-based Bio–NIPU resin. This is also in line with the EDX analysis (Figure 11h), which showed that ramie fiber without the impregnation treatment contained 0% nitrogen (N), while an increase in the N content was seen in the impregnated ramie fiber. This is a result of urethane groups, which were found in lignin-based Bio–NIPU resins and which absorb better with increasing impregnation time, as seen in the percentage weight gain (Table 5) and higher intensity of urethane groups with increasing impregnation time.

The amount of N content produced in the two formulas tended to be the same. This is because the viscosity values of the two lignin-based Bio–NIPU resins were not much different, which is still in the range of 200–300 mPa·s [55]. This is in line with the results of weight gain, where the formula treatment had no significant effect. In addition, the EDX analysis also revealed the presence of sodium (Na) in lignin-based Bio–NIPU-impregnated ramie. This was due to the use of NaOH in the preparation of lignin solutions during the synthesis of Bio–NIPU.

#### 3.3.7. Mechanical Properties Analysis Using UTM

Ramie fiber strength was evaluated using mechanical property analysis. Ramie fiber was tested both before and after impregnation with three different impregnation times. Tensile strength and modulus of elasticity (MOE) are two mechanical strength metrics. For the purpose of choosing the fundamental materials to be used in the creation of functional textiles, it is necessary to be aware of a material’s mechanical qualities.

**Figure 12:**
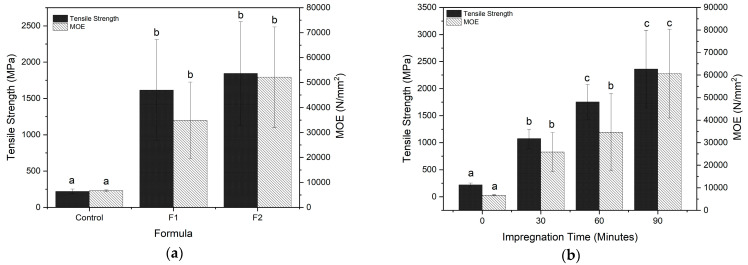
Effect of (**a**) Formula; and (**b**) Impregnation time on the mechanical value of ramie fiber before and after impregnation using lignin-based Bio–NIPU. The values with similar letter (a–c) are not siginificantly different.

The internal structure and morphological traits of the ramie fiber, as well as its chemical composition, are related to its mechanical capabilities, such as its tensile strength. These fibers contain cellulose and high microfibril angles, so they tend to have high tensile strength values compared to other fibers [56]. Ramie fiber’s tensile strength expanded from 221.6 MPa before impregnation to 984.8–2519 MPa after impregnation (Figure 12).

The duration of the impregnation time and the tensile strength of ramie fiber both grew over time. The impregnation period had a substantial impact on the tensile strength of ramie fiber, according to the findings of the analysis of variance (ANOVA) with a 95% confidence interval. Meanwhile, even though there was a slight difference between the formulas, the treatment of formula administration was not significantly different. This can be influenced by the viscosity values of the two impregnant formulas, which are almost the same so that the impregnation ingredients that enter the fiber are also almost the same [17]. This is also in accordance with the WPG data, which has a significant effect only on the addition of impregnation time. The highest tensile strength value was obtained by F2 Bio–NIPU-impregnated ramie with a value of 2519 MPa. This can be influenced by the formation of crystallinity in this formula, which is also higher compared to other treatments. A material’s tensile strength increases with increased crystallinity [54]. The presence of urethane groups in Bio–NIPU, which have been used to create chain bonds and cross-links in Bio–NIPU resin and ramie fiber, is another factor contributing to the increase in tensile strength of ramie fiber.

The modulus of elasticity (MOE) is a measure of a material’s stiffness. The proportional slope of the stress and strain curves’ linear line is known as MOE. The MOE is one of the fundamental parameters in structural design to determine strain and displacements [57]. According to the results obtained, the MOE values followed a similar trend to the tensile strength of ramie fiber. The longer impregnation times resulted in increased MOE values of ramie fiber (Figure 12). Ramie fiber’s tensile strength increased from 6677.68 N/mm^2^ before impregnation to 25,836.37 N/mm^2^ after impregnation to 71,892.47 N/mm^2^ after impregnation. Results of analysis of variance (ANOVA) with 95% confidence interval showed that the impregnation time had a significant effect on the MOE of ramie fiber. Meanwhile, even though there was a slight difference between the formulas, the treatment of formula administration was not significantly different. The highest MOE value was obtained by F2 Bio–NIPU-impregnated ramie with a value of 71,892.57 N/mm^2^. According to [56], relative humidity, diameter length, fiber microstructure, moisture content, and drying technique all have an impact on a material’s mechanical properties.

#### 3.3.8. Ramie Fiber Hydrolysis Analysis

**Figure 13:**
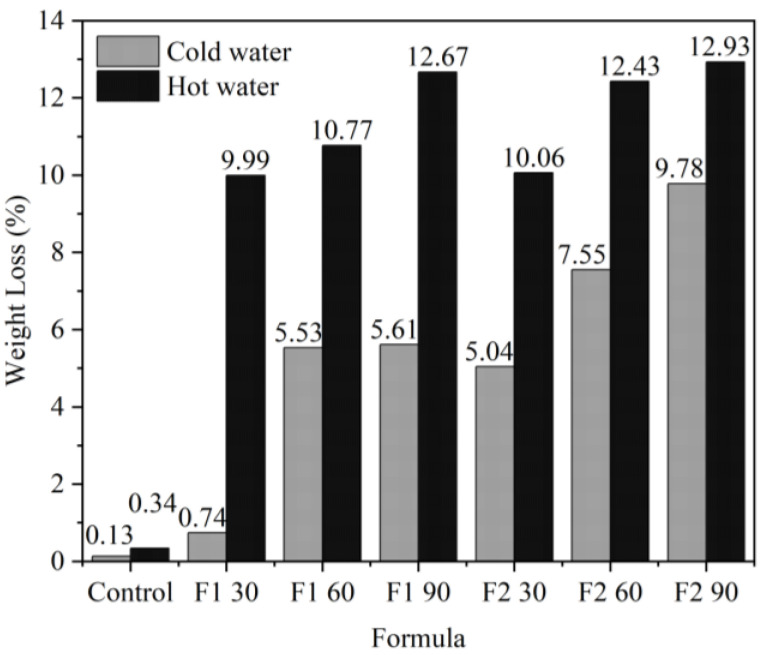
Weight loss of impregnated ramie fibers after washing with cold and hot water.

**Figure 14:**
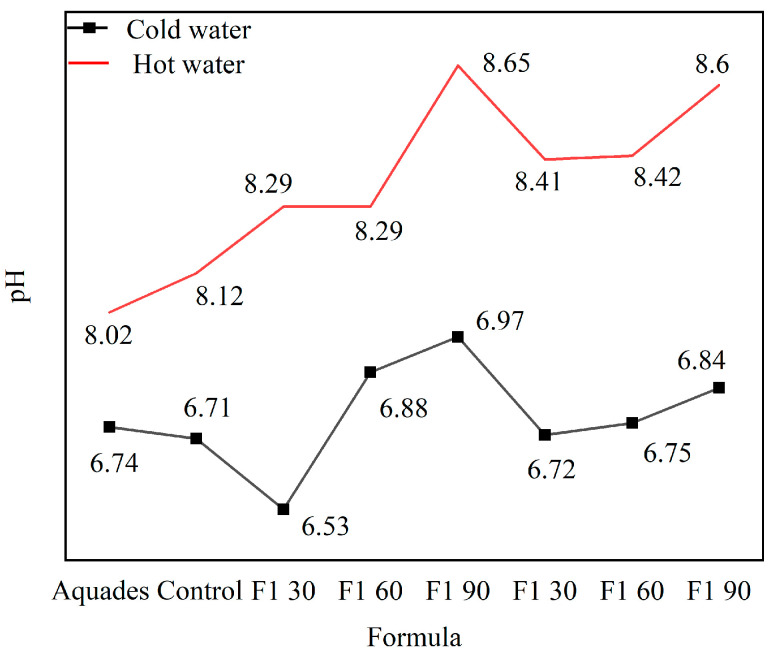
pH value of impregnated ramie fibers after washing with cold and hot water.

The durability of the impregnation to washing was tested using a hydrolysis test on impregnated ramie fiber. Washing-impregnated ramie fiber using cold water did not decrease much in weight (0.74–9.78%) (Figure 13). This is also supported by the pH of the water used for washing, which did not change much in all treatments (Figure 14), and the changes in the water used for washing did not occur significantly. When washing using hot water, a greater decrease in weight in the range of 9–12% was determined. There was a slight change in the pH of the water used for immersion at 60 °C, where the change in pH towards alkaline was caused by Bio–NIPU, which is also alkaline. Therefore, it is recommended in its application to use washing with cold water.

Functional group analysis using FTIR was conducted to determine any changes in functional groups of impregnated ramie fiber after washing using distilled water at different temperatures (cold and 60 °C). Functional groups of impregnated ramie fibers soaked in cold water for one hour had almost the same pattern and intensity as prewashed ramie (Figure S7). This is also supported by the very small value of the percentage of weight loss in cold water hydrolyzed ramie and the water used for washing, which does not often change color. Differences in the absorption pattern and intensity are seen in 60 °C water-hydrolyzed ramie fiber. Only F1- and F2-impregnated ramie (90 and 60 min) were impregnated at wave number 1700 cm^−1^, respectively. Only the impregnated ramie of F1 90, F1 60, and F2 90 showed the peak of wave number 2900 cm^−1^ (N-H). In F2 30-impregnated ramie, the peak at wave number 1500 cm^−1^ (C-N) was not visible. During impregnation time, the bond formed will be stronger. The large percentage of weight loss in impregnated ramie for 90 min is thought to be due to the bonding that occurs not only chemically but also physically, so that it is easily dissolved by the washing solution.

## 4. Conclusions

Black liquor isolation produced lignin with relatively the same characteristics as standard lignin, with better thermal properties. The reaction between lignin–DMC and HMT managed to form an absorption band of the urethane group (R–N–H–(C=O)–R). F2 Bio–NIPU provided the best thermal stability. Bio–NIPU was successfully impregnated into ramie fiber. The longer impregnation times resulted in a better WPG value, FTIR group intensity, thermomechanical properties, crystallinity, and mechanical properties of ramie fiber. F2 Bio–NIPU impregnated hemp fiber based on lignin (Lignin-DMC: HMT (1:2)) after 90 min, which was better than F1 (Lignin-DMC: HMT (1:1)). However, the use of DMC and HMT cannot replace the role of isocyanates in the synthesis of Bio–NIPU because it produces lower heat resistance than pMDI-impregnated ramie. Based on the results obtained, it can be concluded that the impregnation of ramie fiber with L-NIPU represents a promising approach to increase its wider industrial application as a functional material. Future research should be focused on the development of optimal Bio–NIPU formulations in order to achieve better performance of impregnated ramie fiber.

## Figures and Tables

**Table 1:** Characteristics of black liquor used in this work.

Parameter	Results	Reference
Moisture content (%)	27.81 ± 1.11	10 [12]
Solid content (%)	76.79 ± 0.64	65–85 [13]
pH	12.14 ± 0.03	12–13 [15]

**Table 2:** Characteristics of lignin isolated from black liquor.

Parameter	Results	Reference
Yield (%)	47.05 ± 1.94	45.769 [21]
Moisture content (%)	5.07 ± 0.71	8.05 [22]
Ash content (%)	0.31 ± 0.19	8.25–19.19 [23]
*Acid-insoluble lignin* (AIL) (%)	82.54 ± 0.96	53.08 [21]
*Acid-soluble lignin* (ASL) (%)	12.77 ± 0.67	7.26 [21]
Purity (%)	95.32 ± 0.61	60.34 [21]

**Table 3:** Temperature and weight loss of lignin using TGA.

Type of Lignin	T_WL10%_ (°C)	T_WL25%_ (°C)	T_WL50%_ (°C)	Weight Lost (%)	Residue (%)
L-Standard	259	366	597	56.49	42.41
L-Isolated	235	347	644	53.75	46.25

**Table 4:** Temperature and weight loss of lignin Bio–NIPU using TGA.

Formula	T_WL10%_ (°C)	T_WL25%_ (°C)	T_WL50%_ (°C)	Weight Lost (%)	Residue (%)
F1	196	299	513	56.49	34.43
F2	116	257	467	53.75	35.92

**Table 5:** Percentage weight gain in ramie fiber after impregnation.

Formulation	Weight Gain (%)		
	Impregnation Time (min)		
	30	60	90
F1	15.95 ± 5.32	31.46 ± 2.16	45.05 ± 3.97
F2	18.39 ± 5.06	32.42 ± 5.72	51.30 ± 2.52
Average value	17.17 ± 5.19 ^a^	31.94 ± 3.94 ^b^	48.18 ± 3.25 ^c^

**Table 6:** Temperature and weight loss of lignin-based Bio–NIPU using TGA.

Formula	T_WL10%_ (°C)	T_WL25%_ (°C)	T_WL50%_ (°C)	Weight Lost (%)	Residue (%)
Control	196	299	513	82.93	17.07
pMDI	303	336	373	79.07	20.93
F1 30	161	296	350	98.66	1.34
F1 60	191	296	350	95.31	4.69
F1 90	78	170	303	92.93	7.07
F2 30	224	300	353	97.72	2.28
F2 60	113	261	338	92.91	2.09
F2 90	84	187	323	92.11	7.89

## Data Availability

The data presented in this study are available on request from the corresponding author.

## Supplementary Materials

The following supporting information can be downloaded at: https://www.mdpi.com/article/10.3390/ma16165704/s1, Figure S1. Thermal stability analysis of isolated lignin and standard lignin using TGA and DTG. Figure S2. Py-GCMS chromatogram of l-isolated and l-standard. Figure S3. Py-GCMS chromatogram of lignin Bio-NIPU. Figure S4. (a) Viscosity; (b) Cohesion; and (c) Relaxation modulus of lignin Bio-NIPU. Figure S5. Thermo-mechanical analysis of lignin L-NIPU. Figure S6. Crystallinity analysis by ramie fiber using XRD (a) Ramie impregnated with F1 lignin-based Bio-NIPU, and (b) Ramie impregnated with F2 lignin-based Bio-NIPU. Figure S7. Changes in functional groups of imprenated ramie fiber treated by hydrolysis using (a) cold water and (b) ater 60 °C.
