# Supplementary material for: Properties of Ramie (Boehmeria nivea (L.) Gaudich) Fibers Impregnated with Non-Isocyanate Polyurethane Resins Derived from Lignin
Source: Materials (Basel). 2023 Aug 20;16(16):5704. doi: 10.3390/ma16165704 (PMC10456696; doi:10.3390/ma16165704)
Supplement: Supplementary file 1 [file materials-16-05704-s001.zip › materials-2489154-supplementary.pdf]

Supplementary Materials: Properties of Ramie (*Boehmeria nivea* (L.) Gaudich) Fibers Impregnated with Non-Isocynate Polyurethane Resins Derived from Lignin

Supplementary of Figures

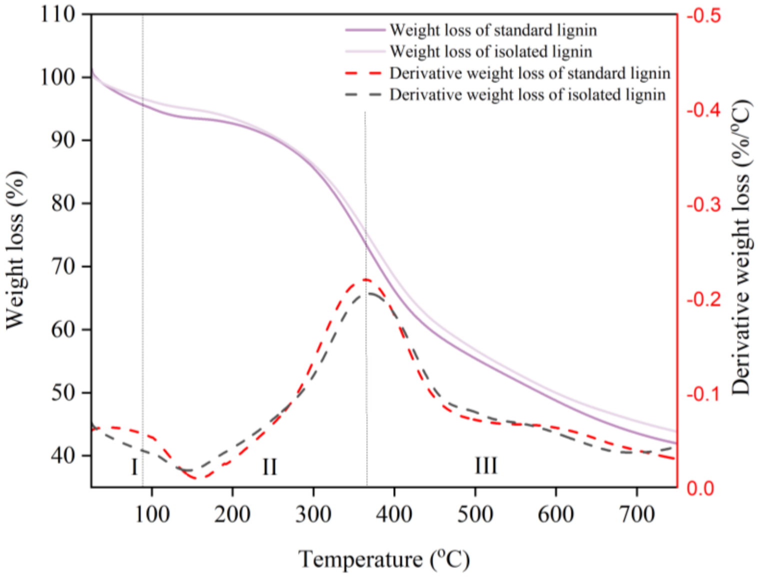

Figure S1. Thermal stability analysis of isolated lignin and standard lignin using TGA and DTG

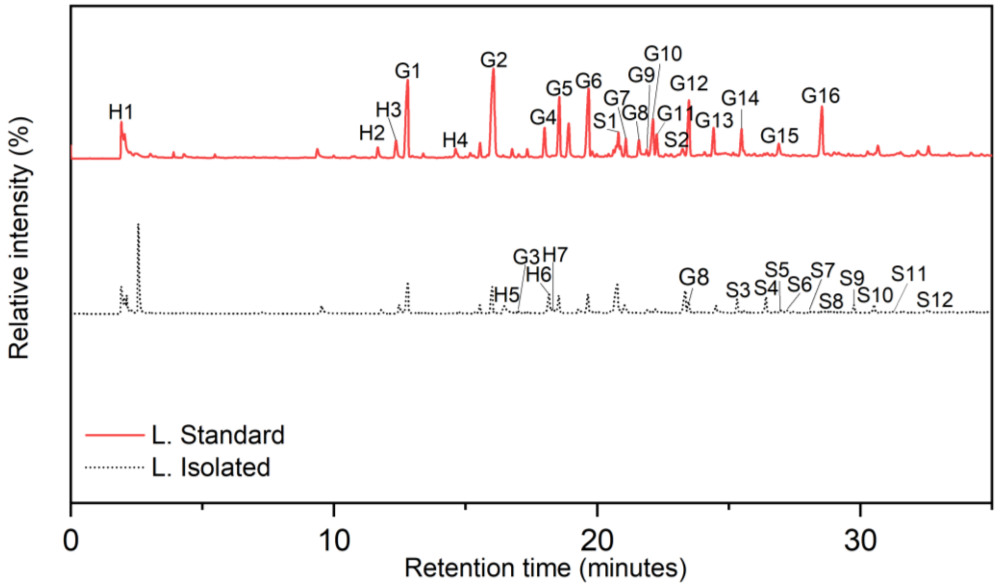

Figure S2. Py-GCMS chromatogram of l-isolated and l-standard.

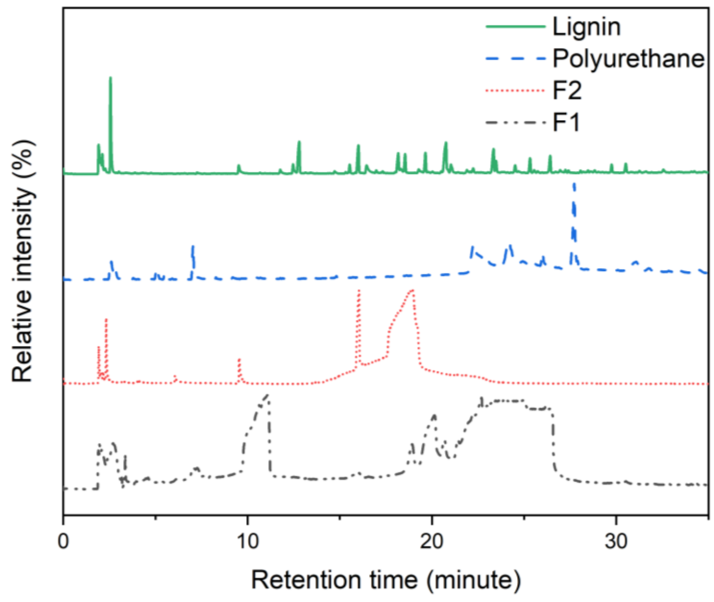

Figure S3. Py-GCMS chromatogram of lignin Bio-NIPU.

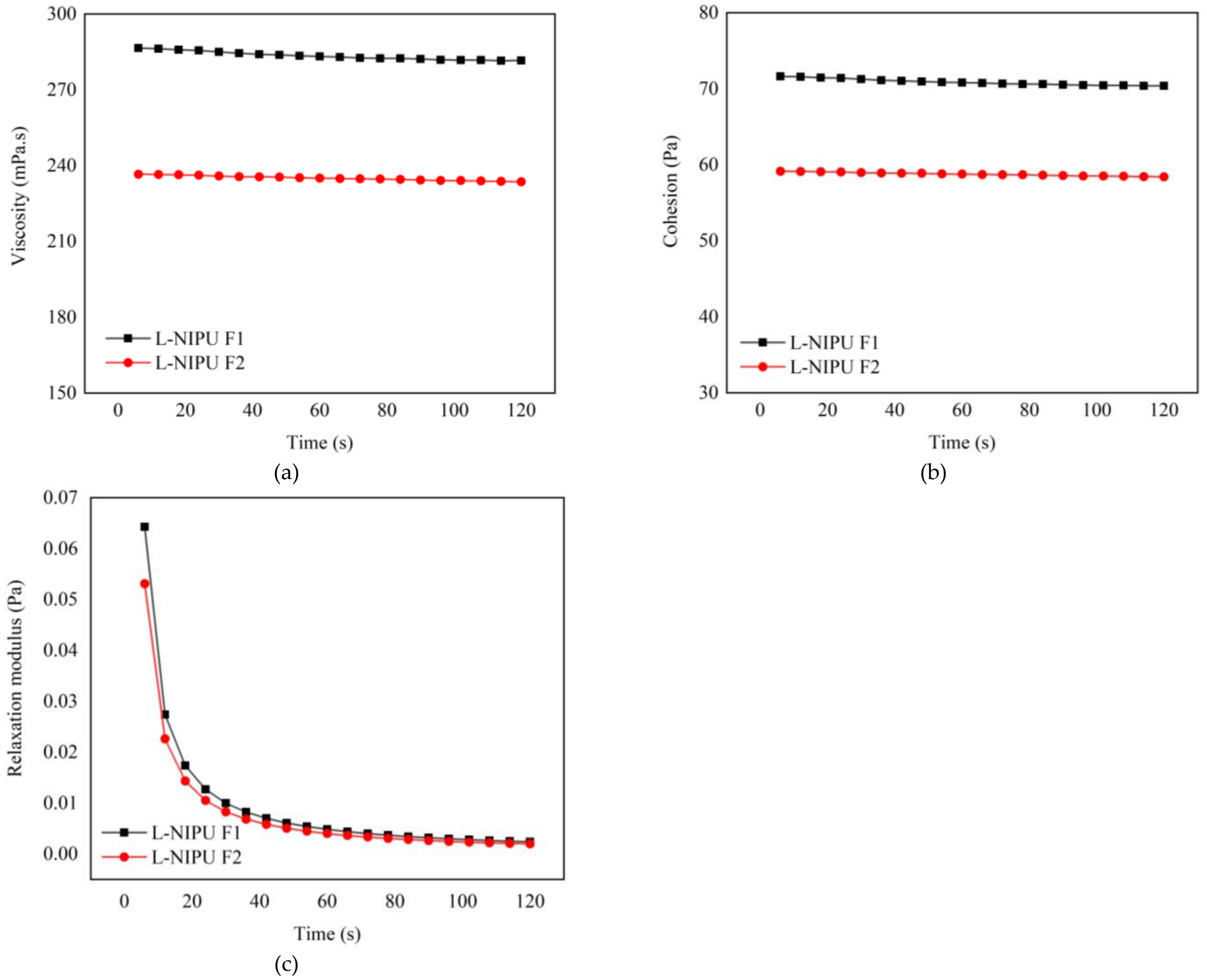

Figure S4. (a) Viscosity; (b) Cohesion; and (c) Relaxation modulus of lignin Bio-NIPU.

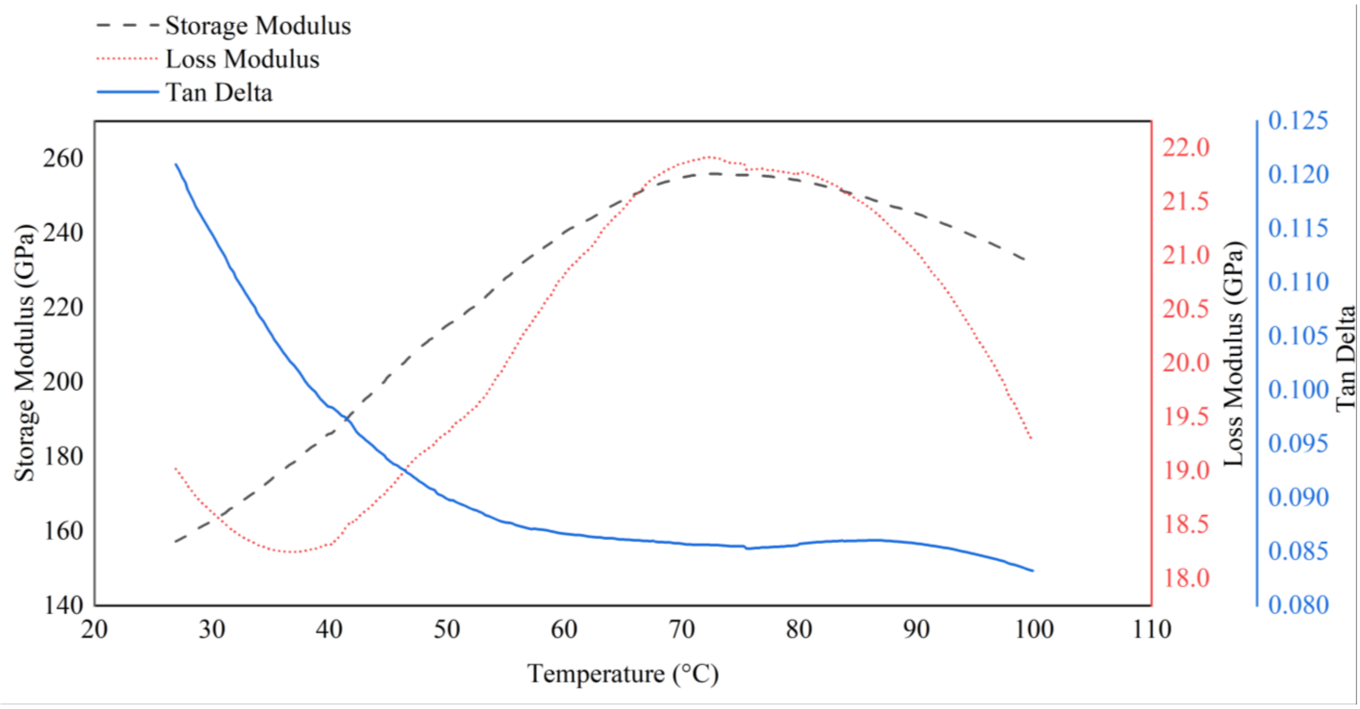

Figure S5. Thermo-mechanical analysis of lignin L-NIPU.

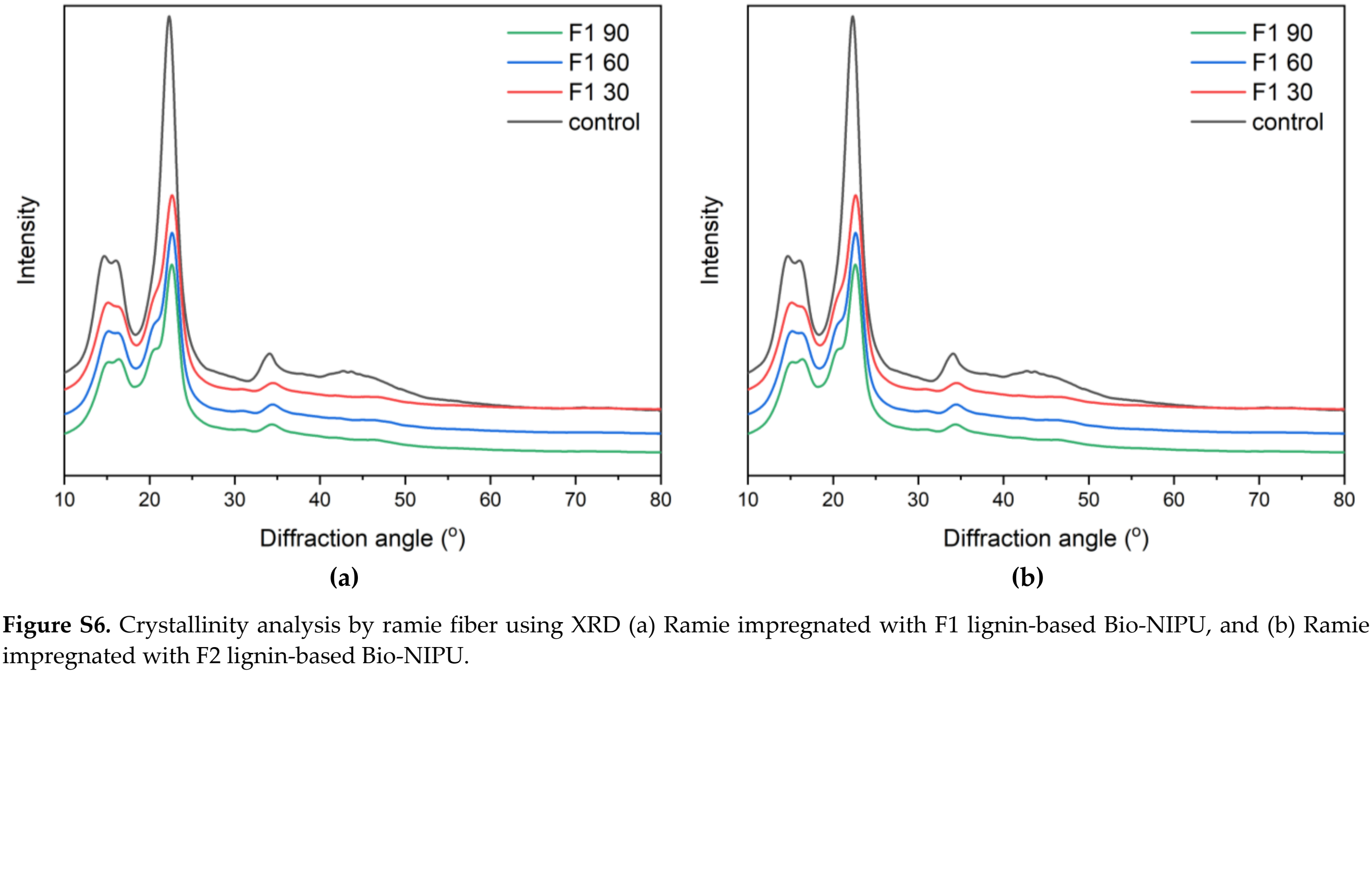

Figure S6. Crystallinity analysis by ramie fiber using XRD (a) Ramie impregnated with F1 lignin-based Bio-NIPU, and (b) Ramie impregnated with F2 lignin-based Bio-NIPU.

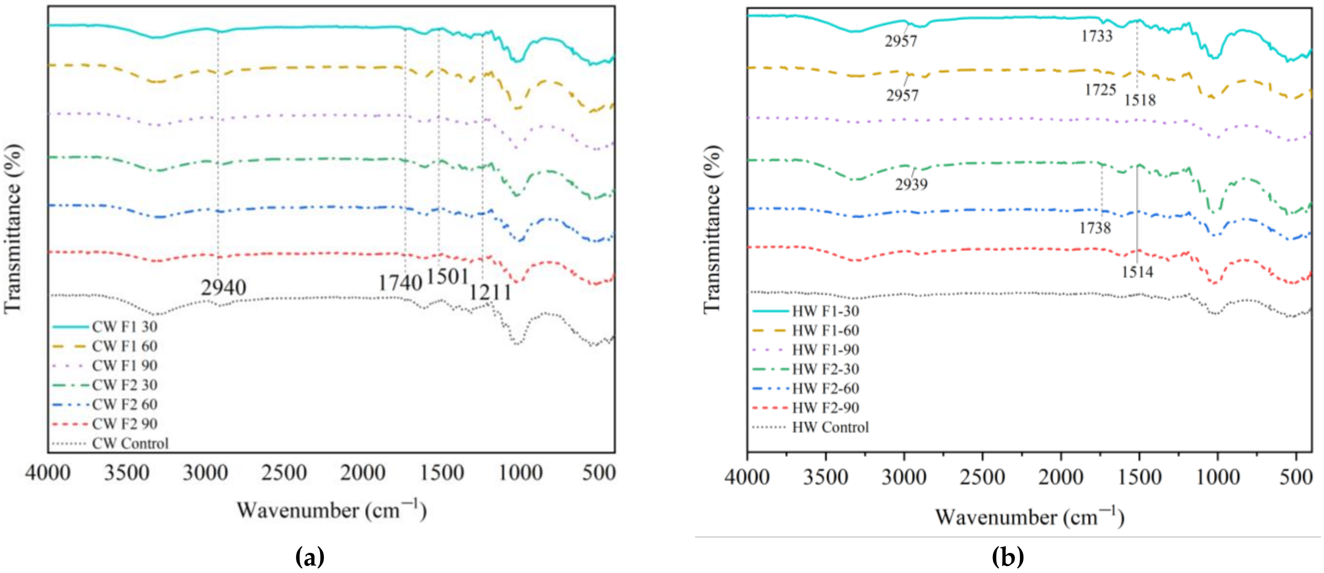

Figure S7. Changes in functional groups of imprenated ramie fiber treated by hydrolysis using (a) cold water and (b) ater 60℃.
